# Constructing arrays of nucleosome positioning sequences using Gibson Assembly for single-molecule studies

**DOI:** 10.1038/s41598-020-66259-4

**Published:** 2020-06-18

**Authors:** Dian Spakman, Graeme A. King, Erwin J. G. Peterman, Gijs J. L. Wuite

**Affiliations:** 1 Department of Physics and Astronomy, and LaserLaB Amsterdam, Vrije Universiteit Amsterdam, De Boelelaan 1081, 1081 HV Amsterdam, The Netherlands; 2 Present Address: Institute of Structural and Molecular Biology, University College London, Gower Street, London, WC1E 6BT UK

**Keywords:** Biophysical methods, Single-molecule biophysics, Nucleosomes, Chromatin, DNA

## Abstract

As the basic building blocks of chromatin, nucleosomes play a key role in dictating the accessibility of the eukaryotic genome. Consequently, nucleosomes are involved in essential genomic transactions such as DNA transcription, replication and repair. In order to unravel the mechanisms by which nucleosomes can influence, or be altered by, DNA-binding proteins, single-molecule techniques are increasingly employed. To this end, DNA molecules containing a defined series of nucleosome positioning sequences are often used to reconstitute arrays of nucleosomes *in vitro*. Here, we describe a novel method to prepare DNA molecules containing defined arrays of the ‘601’ nucleosome positioning sequence by exploiting Gibson Assembly cloning. The approaches presented here provide a more accessible and efficient means to generate arrays of nucleosome positioning motifs, and facilitate a high degree of control over the linker sequences between these motifs. Nucleosomes reconstituted on such arrays are ideal for interrogation with single-molecule techniques. To demonstrate this, we use dual-trap optical tweezers, in combination with fluorescence microscopy, to monitor nucleosome unwrapping and histone localisation as a function of tension. We reveal that, although nucleosomes unwrap at ~20 pN, histones (at least histone H3) remain bound to the DNA, even at tensions beyond 60 pN.

## Introduction

The eukaryotic genome is highly compacted within the nucleus as a result of the formation of chromatin. The basic unit of chromatin is the nucleosome, which consists of 147 base pairs of DNA wrapped approximately 1.7 times around an octamer of histone proteins, resulting in an inner and outer super-helical wrap of the nucleosomal DNA^1,2^. The histone octamer is composed of four homodimers of the histone core proteins H2A, H2B, H3 and H4. The highly positively charged histone octamer binds to DNA via strong electrostatic interactions, resulting in the occlusion of one complete face of the DNA helix from its environment^1^. Consequently, nucleosomes dictate the accessibility of the genome to proteins, and in this way affect essential genomic transactions such as DNA transcription, replication and repair^3,4^. The eukaryotic cell can locally regulate the accessibility of its genome by (de)stabilising specific nucleosomes. Such changes can be achieved via post-translational modifications of histones and/or ATP-dependent chromatin remodeling enzymes^5,6^. Other proteins, such as pioneer transcription factors, HMGB proteins, histone chaperones and RNA polymerase II, have also been reported to alter genome accessibility by interacting with nucleosomes^7,8^.

In recent decades, a range of biochemical and structural studies have provided important insights into the pathways by which different subfamilies of chromatin remodelers and other proteins (de)stabilise nucleosomes^5,7,9–11^. Nevertheless, dynamic and kinetic information on protein-nucleosome interactions has been difficult to extract using such methods. For this reason, *in vitro* single-molecule techniques are becoming increasingly employed to study the interactions of DNA-processing enzymes with nucleosomes and/or chromatin^12–17^. Methods such as atomic force microscopy, Förster resonance energy transfer, DNA curtains, tether particle motion, and magnetic and optical tweezers allow for the possibility to observe dynamic events in real-time, and identify population heterogeneities^16^. Many of these techniques have been exploited with great effect to measure the mechanical stability of both single nucleosomes and nucleosome arrays,^2,18–36^ as well as probe the influence of remodeling and other enzymes on such substrates^8,12–15,17,36–43^.

In order to study nucleosome interactions *in vitro*, it is often desirable to employ a substrate that contains a series of nucleosomes at well-defined positions along the DNA. To this end, nucleosome positioning sequences, which exhibit a high affinity for histone octamers, are often used. DNA sequences that stabilise nucleosomes *in vivo* have been found to be enriched in certain dinucleotide and trinucleotide arrangements (typically ten base pair repeats of TA dimers) that facilitate bending of the DNA double-helix around the histone octamer core^44–46^. Additionally, an extensive screen for sequences that can stabilise nucleosomes was performed by Lowary and Widom, which led to the discovery of the ‘601’ sequence^47^. This artificial sequence has a higher histone affinity than other positioning sequences, such as the native 5S sequence,^47^ and has become widely used for studies of nucleosome structure and function *in vitro*^10,13,23–30,32–36,38,39,43,48–51^. Importantly, the unwrapping of the inner turn of nucleosomes associated with the 601 sequence occurs at similar forces as those measured for native nucleosomes from yeast^52^. This suggests that the 601 sequence is an appropriate model system for studying the interactions of nucleosomes at the single-molecule level. The complete 601 sequence, as defined by Lowary and Widom,^47,53^ consists of 282 base pairs, of which 147 bear a high affinity for the histone octamer. These 147 base pairs are referred to as the 601-core sequence. The additional 135 base pairs form two nucleosome-free linker regions that flank either side of the 601-core. A number of strategies have been used to construct DNA substrates containing arrays of up to 72 601 motifs^48,54–56^. Typically, these arrays consist of repeats of the 601-core sequence flanked by linker DNA that is derived from the 135 base pairs of linker regions associated with the complete 601 sequence. In contrast to mono-nucleosomes, an array of nucleosomes facilitates studying chromatin-like structures,^25,41,48–50,55,57^ and can be used to probe the influence of closely spaced nucleosomes on DNA-protein interactions^15,36,40,41^.

In recent years, several strategies to generate arrays of the 601 nucleosome positioning motif have been reported. One widely-used approach, described by Huynh *et al*.,^55^ is to self-ligate fragments that each contain a single 601 motif. This results in a linear array of 601 repeats that can then be embedded in a suitable plasmid. With such an approach, however, it can be difficult to pre-determine how many repetitive motifs will be generated during the ligation process, which substantially reduces the ease with which a library of plasmids with different 601 repeats can be generated. To overcome this, Wu *et al*.^56^ and Dyer *et al*.^54^ have devised strategies involving sequential addition of 601 fragments to a plasmid, where the plasmid acts as both a vector (into which 601 repeats can be embedded) and a host (from which 601 repeats can be extracted). In this way, a library of plasmids with defined arrays of 601 motifs can be generated. Nevertheless, generating substrates containing 601 arrays can still be time-consuming and often requires careful design of target sequences flanking the 601 motifs.

Here, we describe a novel method to construct DNA substrates with multiple repeats of nucleosome positioning sequences in an efficient and controlled manner by exploiting Gibson Assembly cloning. Since its inception in 2009, Gibson Assembly has revolutionised molecular cloning by enabling efficient joining of multiple DNA fragments in a single isothermal reaction^58,59^. In addition to the intrinsic benefits of using Gibson Assembly, our bespoke design of ‘Gibson Inserts’ offers straightforward control over both the number of nucleosome positioning motifs and the linker sequences flanking these repeats. Moreover, we show that arrays of the 601 motif generated via this approach are well suited for combined single-molecule manipulation and fluorescence imaging studies. In particular, we reveal that histones (at least histone H3) remain bound to the DNA following force-induced nucleosome unwrapping even under high (≥60 pN) tensions.

## Results and Discussion

### **A Gibson Assembly based strategy for constructing nucleosome positioning arrays**

**Figure 1:**
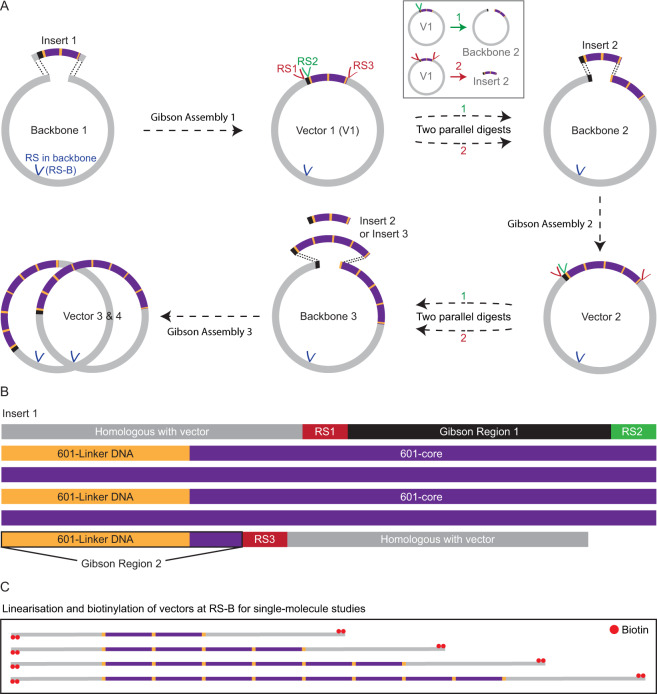
Schematic representation of a Gibson Assembly based cloning strategy for obtaining arrays of 601 nucleosome positioning motifs. **(A)** Insertion of a segment containing *n* × 601 repeats into a linearised plasmid via sequential Gibson Assembly reactions. In the first Gibson Assembly reaction, a fragment containing two 601-core repeats flanked by identical linker sequences (Insert 1) is embedded in a suitable plasmid (Backbone 1). The resulting vector (Vector 1) can then be used to obtain a new insert (Insert 2) containing two 601 motifs via digestion at restriction sites RS1 and RS3 (Inset). In a parallel reaction (Inset), Vector 1 can also be digested at restriction site RS2 to yield a backbone containing two 601 repeats into which Insert 2 can be embedded via a Gibson Assembly reaction. This procedure can be repeated until the desired number of 601 motifs has been obtained. **(B)** Sequence composition of Insert 1. Two 601-core repeats (corresponding to the 147 base pairs of the 601-core sequence, purple) are flanked by identical linker sequences (yellow). The ends of Insert 1 (grey) are homologous with the ends of Backbone 1 to facilitate the first Gibson Assembly reaction. Additionally, Insert 1 contains two ‘Gibson regions’ (Gibson Region 1 and Gibson Region 2), as well as three restriction sites (RS1, RS2, and RS3), designed in such a way that, once Insert 1 has been incorporated into Backbone 1, further 601 motifs can be embedded via subsequent Gibson Assembly steps (as shown in panel A). **(C)** The library of plasmids containing *n* × 601 repeats prepared using the approach outlined in panel A can be used directly for single-molecule studies after linearisation and biotinylation at an appropriate restriction site (RS-B in panel A).

Our goal is to devise an efficient procedure to generate a DNA substrate containing a defined array of nucleosome positioning sites. To this end, we exploit the Gibson Assembly cloning method^58^ to sequentially insert short DNA segments containing a given number of 601-core nucleosome positioning sequences, each separated by a defined linker length, into a longer plasmid. This is summarised in Fig. 1A. In this procedure, a segment (Insert 1, Fig. 1B) containing two 601-core sequences each flanked by 25 base pairs of identical linker DNA was first generated by ligating two readily synthesisable DNA constructs (Methods and Supplementary Fig. S1). Insert 1 can then be embedded in a linearised plasmid (Backbone 1) through a Gibson Assembly reaction. In order to facilitate this reaction, Insert 1 contains two regions of 40 base pairs at both of its ends that are homologous to the terminal sequences of Backbone 1 (shown in grey in Fig. 1A,B). Insertion of Insert 1 into Backbone 1 thus yields a plasmid containing two 601 motifs (Vector 1). Insert 1 also contains three restriction sites (RS1–3) and two ‘Gibson regions’ of 33 base pairs (Gibson Region 1) and 32 base pairs (Gibson Region 2), respectively (Fig. 1B). These are designed such that arrays of multiple 601 motifs can be constructed in the following manner. To obtain an array of four 601 repeats, Vector 1 is used as a template for two parallel digestion reactions. In the first reaction, Vector 1 is digested at RS1 and RS3, resulting in a segment (Insert 2) that contains two 601 motifs. In a second parallel reaction, Vector 1 is digested at RS2 to yield a linearised vector containing two 601 motifs (Backbone 2). The ends of Insert 2 (Gibson regions) are each homologous to the terminal regions of Backbone 2. In this way, a Gibson Assembly reaction of Insert 2 and Backbone 2 yields a vector containing an array of four 601 motifs (Vector 2). The above procedure can be repeated until the desired number of 601 repeats has been obtained (Fig. 1A). After each Gibson Assembly reaction, the resulting vector can be linearised and biotinylated for single-molecule studies (Fig. 1C).

### **Experimental characterisation of a library of nucleosome positioning arrays**

**Figure 2:**
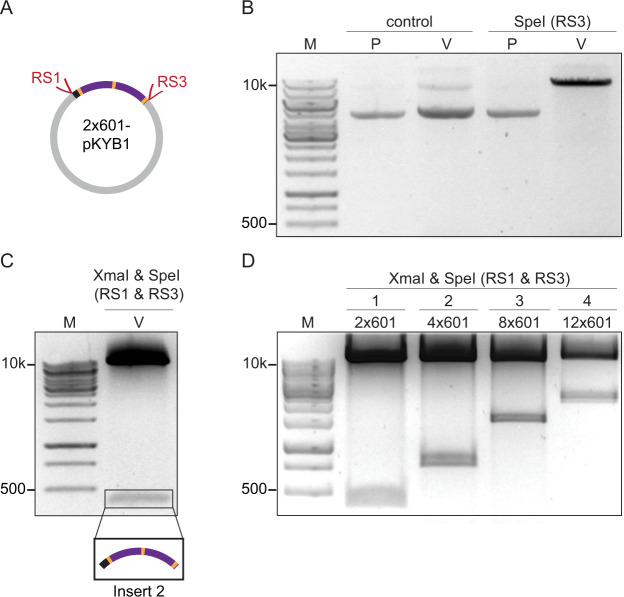
Generation of inserts and plasmids containing arrays of *n* × 601 motifs obtained after sequential Gibson Assembly reactions. **(A)** Schematic illustration of Vector 1 (*cf*. Fig. 1A), consisting of two 601-core repeats (purple) flanked by 25 base pairs of linker DNA (yellow) embedded within a pKYB1 plasmid (resulting in a 2 × 601-pKYB1 vector). **(B)** Plasmids were screened for successful cloning by digestion at restriction site RS3 with SpeI and analysed by agarose gel electrophoresis (0.6% agarose gel), as shown here for one representative plasmid. Lanes 2 and 3 correspond to pKYB1 (P) and Vector 1 (V), respectively, while Lanes 4 and 5 correspond to the same plasmids following SpeI treatment. Only Vector 1 is digested by SpeI, confirming that this plasmid corresponds to the 2 × 601-pKYB1 vector. **(C)** The 2 × 601-pKYB1 plasmid is digested at restriction sites RS1 (with XmaI) and RS3 (with SpeI) to yield a 2 × 601 segment of 417 base pairs (Insert 2, *cf*. Fig. 1A). This segment was identified using agarose gel electrophoresis (1.5% agarose gel). **(D)** Agarose gel electrophoresis (1.5% agarose gel) of segments containing two, four, eight and twelve 601 motifs, digested from four different plasmids following four sequential Gibson Assembly reactions (labelled 1–4), as described in Fig. 1A.

To verify the robustness of the above procedure, we first embedded a construct containing 2 × 601 motifs (Insert 1) in a linearised pKYB1 plasmid (*cf*. Fig. 1A) via a Gibson Assembly reaction (Fig. 2A). The resulting plasmids were screened for successful cloning by digestion at RS3, followed by agarose gel electrophoresis (Fig. 2B). All clones carried a vector containing two 601 motifs (2 × 601-pKYB1), from which a further insert (Insert 2, *cf*. Fig. 1A) containing two 601 motifs was obtained by digestion at RS1 and RS3 (Fig. 2C). Embedding Insert 2 in the previously prepared 2 × 601-pKYB1 vector via Gibson Assembly, yielded a vector containing four 601 motifs (4 × 601-pKYB1), from which a third insert (Insert 3, *cf*. Fig. 1A) containing four 601 motifs was obtained (Fig. 2D). By embedding either the 2 × 601 insert or the 4 × 601 insert in a 4 × 601-pKYB1 or 2 × 601-pKYB1 plasmid via a third Gibson Assembly reaction, vectors containing even more 601 motifs can be generated. We chose to embed the insert with four 601 motifs (Insert 3) in the 4 × 601-pKYB1 backbone to obtain a plasmid containing eight 601 motifs. Following this, we inserted the 4 × 601 insert into the previously prepared 8 × 601-pKYB1 vector via a fourth Gibson Assembly reaction to yield a plasmid containing twelve 601 repeats. In this way, a library of plasmids containing a range of 601 motifs was generated (Fig. 2D).

### **Construction of nucleosome positioning arrays with variable linker lengths**

**Figure 3:**
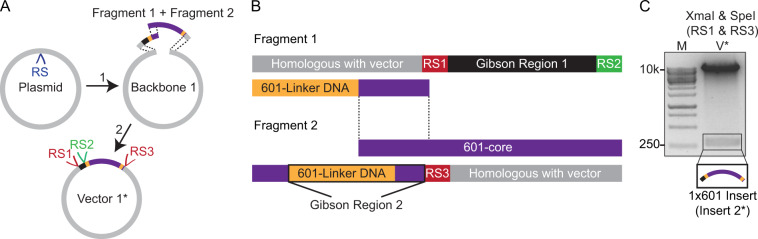
Generation of plasmids containing integer numbers of 601 repeats with linker lengths of any size via Gibson Assembly reactions. **(A)** Insertion of a single 601 motif into a linearised plasmid via a three component Gibson Assembly reaction. Here, two DNA fragments (Fragment 1 and Fragment 2), that together form a single 601-core flanked by identical 601-linker sequences, are embedded in a suitable plasmid (Backbone 1). **(B)** Sequence composition of Fragments 1 and 2. Fragment 1 contains a 40 base pair region (purple) that is homologous to the 5’ end of Fragment 2 (highlighted by the dashed lines). Additionally, Fragments 1 and 2 each contain a 40 base pair region homologous to the 3’ and 5’ ends of Backbone 1, respectively (grey). These homologous regions enable the Gibson Assembly reactions depicted in panel A. Furthermore, Fragment 1 contains a Gibson Region 1, as well as two restriction sites (RS1 and RS2), while Fragment 2 contains a Gibson Region 2 and one restriction site (RS3). These are designed in such a way that, once both Fragment 1 and Fragment 2 have been incorporated into Backbone 1, further 601-based inserts can be generated by digestion of Vector 1* followed by Gibson Assembly reactions (analogous to that shown in Fig. 1A). **(C)** A pKYB1 plasmid containing a single 601-core sequence flanked by linker sequences of 50 base pairs was prepared using the approach outlined in panel A. This vector (V*) was digested at restriction sites RS1 (with XmaI) and RS3 (with SpeI) to yield a 1 × 601 segment (Insert 2*) of 303 base pairs. This segment was identified using agarose gel electrophoresis (1.5% agarose gel) and can be used for further Gibson Assembly reactions (*cf*. Fig. 1A).

Insert 1, as defined in Fig. 1, is limited to linker lengths less than ~30 base pairs owing to the technical difficulties in synthesising long tracts of identical linker sequences (Supplementary Methods). Nevertheless, linker lengths <30 base pairs have been reported to exist *in vivo*, depending on cell type and genomic position,^60–64^ and are thus frequently used for *in vitro* measurements^28,34,41,49–51^. However, linker lengths ≥ 30 base pairs can additionally arise *in vivo,*^61,63,65,66^ and can therefore also be relevant for *in vitro* studies^15,25,27,28,35,38,39,41,49–51^. Such longer linker lengths can be engineered using our approach by simply modifying the first step of the strategy laid out in Fig. 1A, as shown in Fig. 3A. Here, two DNA fragments (Fragments 1 and 2), which together form a single 601-core flanked by identical 601-linker sequences, are embedded in a relevant plasmid via a single Gibson Assembly reaction (Fig. 3A). Since each fragment contains only a single linker sequence (and is thus free from extensive repetitive sequences), much longer linker lengths can be engineered (Supplementary Methods). As shown in Fig. 3B, Fragments 1 and 2 together contain three restriction sites (RS1–3) and two ‘Gibson regions’, analogous to Insert 1 in Fig. 1B. This enables a segment containing a single 601 motif (denoted here as Insert 2*) to be extracted from the 1 × 601 plasmid and used for subsequent Gibson Assembly reactions, following the general strategy laid out in Fig. 1A. To validate this, a variant of Insert 2* containing 50 base pair linkers was extracted from a 1 × 601-pKYB1 vector using the above approach (Fig. 3C). In this way, a library of plasmids can be generated with integer numbers of 601 repeats (including *n* = 1) and, importantly, with linker lengths of any size. This latter approach, in which an initial 1 × 601 vector is used to generate higher order arrays, is therefore highly versatile. Nevertheless, when constructing arrays of even numbers of the 601 motif with linker lengths <30 base pairs, it is both cheaper and more time efficient to begin the procedure with a 2 × 601 insert (as illustrated in the first step of Fig. 1A).

### **Advantages of our Gibson Assembly based approach**

The self-cycling reactions described above, where the plasmid acts as both a vector (into which 601 repeats can be embedded) and a host (from which 601 repeats can be extracted), are broadly similar to the methods reported previously by Wu *et al*.^56^ and Dyer *et al*.^54^. However, our approach provides a number of distinct advantages. Most notably, our methods exploit Gibson Assembly reactions, which are superior in this context for several reasons. First, Gibson Assembly is very fast: insertion of a 601-based fragment into a vector takes around one hour. Second, it is highly efficient: each step of our reactions has near 100% efficiency, whereas efficiencies of ~40% have been reported previously for a 16 × 601 array^54^. Third, Gibson Assembly can typically be performed with lower amounts of sample than standard ligation reactions, reducing the requirements for large quantities of starting reagent. As well as our use of Gibson Assembly, our strategies offer a number of other important features. First, in the approach outlined in Fig. 1, an array of many 601 repeats can be generated in very few steps, owing to the fact that a fragment containing two 601 repeats is used as the initial insert (Supplementary Fig. S1). Second, in the approach described in Fig. 3, no restriction sites are required to be positioned within the linker sequences. Third, in the approaches in both Figs. 1 and 3, all linker sequences flanking the 601 cores can be made identical. The latter two features ensure full control over the length and sequence of the linker regions. Nucleosomes have been reported to slide or diffuse in a sequence-dependent manner,^67^ and such dynamics can be modulated or promoted by chromatin-associating proteins^41^. The integrity of the 601-linker sequences could potentially be important when studying such effects and therefore it is desirable to ensure the linker sequences are all identical and contain no interruptions.

### **Combined single-molecule manipulation and fluorescence imaging of nucleosome arrays**

Using the approaches outlined above, defined arrays of nucleosomes can be reconstituted and studied *in vitro*. Knowledge of the mechanical properties of such nucleosome arrays is vital for understanding the fundamental interactions of chromatin and how these are regulated by chromatin-associating proteins^68–71^. In particular, arrays of regularly positioned nucleosomes are ideally suited for interrogation with single-molecule approaches. In such studies, long nucleosome-free handles are advantageous as they minimise sticking of nucleosomes to the surface or bead that the DNA is tethered to. Furthermore, long handles on either side of a nucleosome array are beneficial for fluorescence imaging, as they minimise interference from the tethered surface or beads. For these reasons, we used a relatively long plasmid (pKYB1, ~8.4 kilo base pairs) as a vector for the cloning steps described above, yielding DNA handles of 3.45 and 4.95 kilo base pairs, respectively, after a single digestion. We demonstrate here that a 12 × 601-pKYB1 construct can be readily exploited to probe the physical properties of a nucleosome array using a combination of optical tweezers and fluorescence microscopy. To this end, a 12 × 601-pKYB1 construct (with 25 base pair linkers) was linearised and biotinylated (Fig. 1C), and nucleosomes were reconstituted on this substrate by gradient salt dialysis.

**Figure 4:**
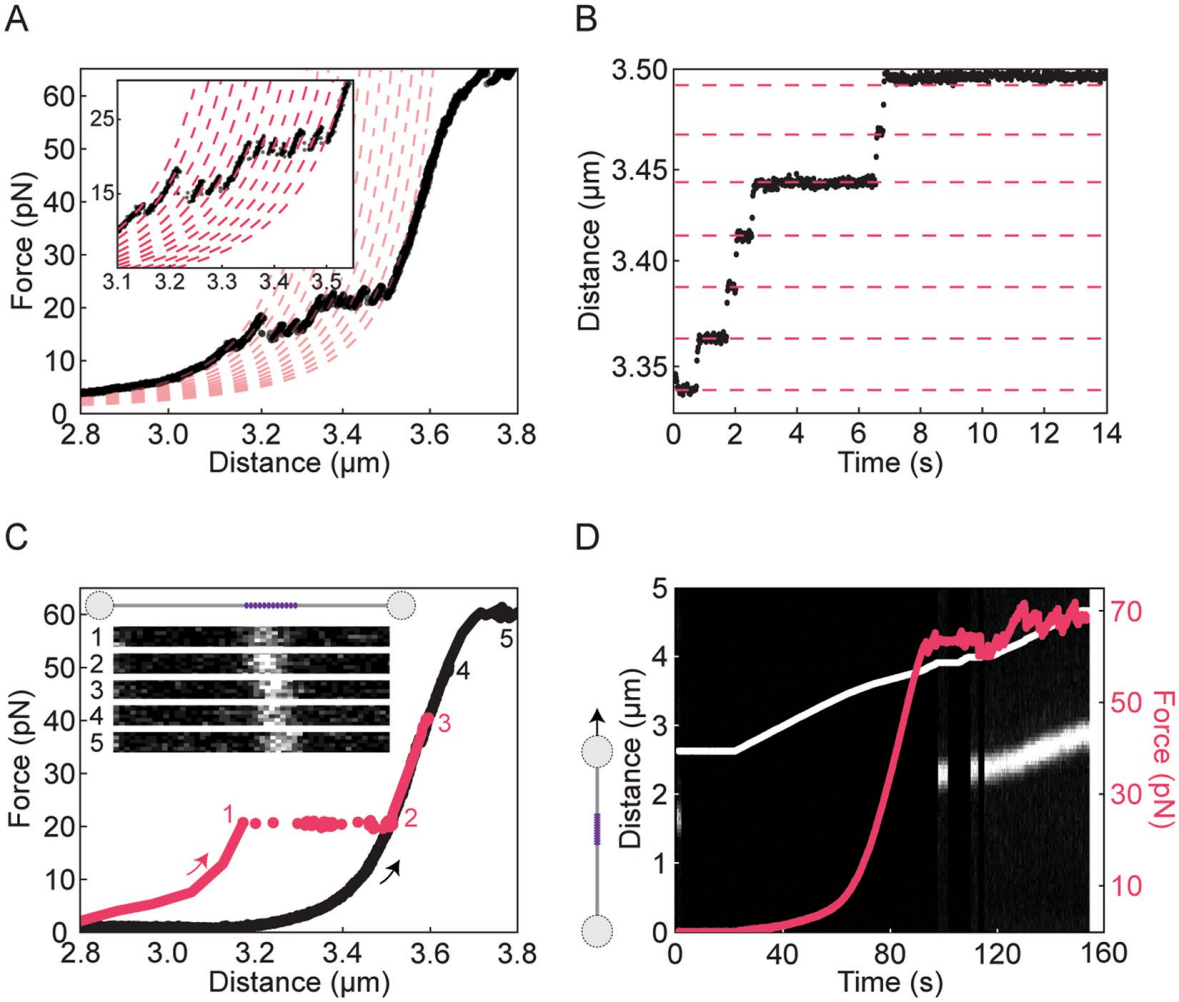
Characterisation of a 12-nucleosome array using dual-trap optical tweezers in combination with confocal fluorescence microscopy. **(A)** A sample force-distance curve of a 12-nucleosome array (derived from a linearised 12 × 601-pKYB1 vector with 25 base pair linkers) recorded using a stretching speed of 20 nm s^–1^. The unwrapping of the inner turn of each of the twelve nucleosomes is identified by the characteristic saw-tooth-like pattern ~20 pN. The expected shift in DNA contour length after each inner turn unwrapping event (~25 nm) is identified by fitting the force-distance data to a series of curves based on the Worm-Like Chain model (dashed red lines). **(B)** DNA extension for the 12-nucleosome array at a constant force of 20 pN as a function of time, showing abrupt step changes of ~25 nm (highlighted by the dashed red lines). These steps correspond to the ~80 base pairs that are released following unwrapping of the inner turn of each nucleosome. **(C)** Sample force-distance curve of the 12-nucleosome array after pre-incubation with Alexa-647-labelled histone H3 antibody. Corresponding confocal fluorescence images (inset) were recorded: (1) immediately after increasing the tension to 20 pN; (2) after 60 seconds at 20 pN; and (3) after 60 seconds at 40 pN. The corresponding change in DNA extension is shown in red. Images 4–5 were recorded at 50 pN and 60 pN, respectively, after 60 seconds at each force. Note that prior to recording images 4 and 5, the DNA molecule was retracted and re-extended (highlighted by the black force-distance curve). **(D)** Sample kymograph of histone H3 antibody fluorescence associated with the 12 × 601-pKYB1 construct as a function of time as the construct is stretched at a speed of 20 nm s^−1^. The corresponding force and extension are overlaid on the kymograph in red and white lines, respectively. Note that in order to ensure all nucleosomes were first unwrapped, the construct was stretched from 0 to 60 pN prior to this measurement. Therefore, the kymograph shown here represents a ‘re-stretch’ following nucleosome unwrapping. In order to minimise bleaching, the fluorescence excitation source was strobed, such that excitation was permitted only at low forces (<1 pN) and at high forces ≥ 60 pN.

The force-extension properties of nucleosome arrays have been the focus of extensive research over the past twenty years. It is well-known that the outer turn of each nucleosome (consisting of ~60 base pairs) unwraps at low forces (∼5pN), while the inner turn unwraps at forces of ~10–40 pN^2,18–21,26,28^. In constant-velocity stretching experiments (*e.g*. using optical tweezers), the latter results in a saw-tooth-like force-extension profile due to the sequential unwrapping of each nucleosome in the array. Each force rupture is roughly evenly spaced by ~25–30 nm corresponding to the ~80 base pairs associated with the inner turn of each nucleosome^2,72–74^. We recapitulate these findings here with our 12-nucleosome array; Fig. 4A shows a typical force-distance curve, exhibiting 12 force ruptures between 10 and 40 pN. In order to monitor the unwrapping of the inner turn in real-time, we also measured the change in DNA extension at a constant tension of 20 pN (Fig. 4B). In both force-extension and constant-force measurements, each unwrapping of the inner turn results in the expected lengthening of ~25 nm (indicated by the red lines in Fig. 4A,B).

It has been shown before that after stretching to ~40–50 pN, nucleosomes can sometimes reform once the tension is reduced to below a few pN^2,18–21,26^. However, the probability of nucleosomes reforming after unwrapping decreases significantly either after repeated stretch-relax cycles^2,19–21^ (see also Supplementary Fig. S2) or after slow stretching (Fig. 4C). It has therefore been suggested that histones may disassemble under applied tension^2^. However, it remains unclear whether histones completely detach from the DNA under mechanical strain or simply cannot reform nucleosomes. An advantage of manipulating nucleosome arrays using optical tweezers is that this technique can be readily combined with fluorescence imaging, allowing nucleosome positioning to be identified directly. We sought to exploit this combined functionality to track histone binding as a function of applied force. To this end, we incubated the above 12-nucleosome array with fluorescently-labelled (Alexa-647) histone H3 antibodies.

We first recorded fluorescence images at a constant force of 20 pN, both before and after nucleosome unwrapping occurred (frames 1 and 2 in Fig. 4C). A further fluorescence image was recorded at 40 pN after waiting for 1 minute at this force (frame 3, Fig. 4C). No significant difference in fluorescence intensity was observed between these images, supporting the hypothesis that histones (at least histone H3) can remain bound to the DNA (after nucleosome unwrapping) up to forces of at least 40 pN. We next reduced the extension and re-stretched the DNA molecule (black trace, Fig. 4C). During this re-stretch, no nucleosome reformation was detected. However, fluorescent H3 antibodies, and thus histone H3, continued to be observed at 50 pN (frame 4, Fig. 4C), and also during DNA overstretching (frame 5 in Fig. 4C and Fig. 4D). These observations were highly reproducible: from a set of twelve nucleosome arrays studied in this way, only a single nucleosome re-wrap was observed to occur after the tension was decreased from 40 pN (*i.e*. 1/144 nucleosomes re-formed; 11 molecules showed no re-wrapping at all); yet fluorescent H3 antibodies were observed on every DNA substrate studied during the re-stretch. Moreover, we observed little difference in the antibody fluorescence intensity between the first and second stretch (Supplementary Fig. S3). Since the number of fluorescent labels on the antibodies varies (Methods), it is not possible to determine the number of antibodies present on a given DNA molecule. Nonetheless, it is clear that at least one antibody is present on every DNA molecule. This suggests that histone H3 does not detach from the DNA even though rewrapping of nucleosomes does not occur. To confirm that these findings were not influenced by the antibody, we repeated the above experiments with a different histone labelling strategy, using Atto-647N-labelled histones (Supplementary Methods). These experiments yielded similar results as before, namely persistent binding of histones well into the overstretching transition (Supplementary Fig. S4). We also note that the average lifetime of a wrapped nucleosome at 20 pN did not change significantly in the presence of either the anti-H3 antibody or Atto-647N dye, indicating that neither fluorescent probe substantially alters nucleosome stability.

Taken together, the above findings indicate that the failure of nucleosomes to reform upon relaxation from high force to low force does not necessarily correlate with complete histone dissociation from the DNA. This raises an intriguing question: why is re-wrapping not always observed when histones are still bound to the DNA? One possibility is that the histone octamer *partially* fragments under tension, preventing nucleosomes from reforming when the tension is lowered. There is evidence that the histone octamer disassembles sequentially under increasing ionic strength, with the H2A/H2B dimers dissociating first, followed by the H3/H4 tetramer^28,30,41,75^. Moreover, it has been suggested that a similar fragmentation process can occur under tension, whereby the H2A/H2B dimers dissociate at lower forces than the H3/H4 tetramer^22,33^. Therefore, it is possible that, even at 20 pN, the histones we observe are only associated with the H3/H4 tetramer. However, H3/H4 tetramers are known to form stable nucleosomes, called tetrasomes, which exhibit similar force-extension properties (between 10 and 40 pN) as canonical nucleosomes^29,31^. Consequently, it would appear likely that any intact H3/H4 tetramer present at high forces should be able to reform nucleosomes upon reduction of the tension. We therefore speculate that after nucleosome unwrapping, the H3/H4 tetramers may no longer remain intact. One possibility is that the H3/H4 tetramer splits into H3/H4 dimers on the DNA. These dimers could each interact with the DNA in such a way that limits their ability to reassemble tetrasomes or nucleosomes upon reduction of the tension, even though the histones remain bound. In support of this, we note that the H3/H4 tetramer can be disrupted by remodeling proteins such as ASF^76^ and there is evidence that the tetramer can split into H3/H4 dimers *in vivo* at transcriptionally active genes^77,78^. We suggest that a similar disruption of H3/H4 tetramers could potentially occur on DNA under sufficient tension. Finally, it is important to note that although tensions of ≥ 60 pN are generally not expected to occur *in vivo*, several chromatin-associating proteins can disrupt nucleosome stability through the generation of torsional strain^5,71^. The persistent binding of at least some histones to the DNA after mechanical disruption could therefore be equally relevant in this context.

## Conclusions

Here, we present an efficient and versatile methodology to generate DNA constructs containing an array of well-defined nucleosome positioning sites by exploiting Gibson Assembly cloning. In this way, we obtain a library of plasmids containing one, two, four, eight and twelve 601 nucleosome positioning sites. With this approach, each plasmid can either be used to generate nucleosome arrays directly or can be exploited to obtain additional inserts and backbones to increase the number of nucleosome positioning sites further, in a highly controlled manner. Moreover, this same procedure could easily be adapted to insert a wide range of other repetitive DNA sequences within a relevant vector, such as the 5S nucleosome positioning sequence or other protein binding sequences. We further show that defined nucleosome arrays, flanked by long nucleosome-free handles, are well suited for combined optical tweezers and fluorescence imaging studies. In this way, we provide the first direct evidence that histones can remain bound to DNA following nucleosome unwrapping, even at forces ≥ 60 pN. We suggest that this may arise from the partial fragmentation of the histone octamer upon stretching.

## Methods

### **Reagents**

All DNA constructs, except for pKYB1, were purchased from Integrated DNA Technologies (Supplementary Table S1). pKYB1, T4 DNA ligase, alkaline phosphatase calf intestinal (CIP), restriction enzymes, Gibson Assembly Master Mix, and SOC outgrowth medium were purchased from New England Biolabs (NEB). Restriction digestions were performed in NEB Cutsmart buffer or NEBuffer 3.1. The GeneRuler 1 kb DNA ladder (Thermo Fisher Scientific) was used to estimate DNA fragment sizes. Digestions were cleaned using the MinElute Reaction Clean-up kit (Qiagen). Gel extraction was performed using the QiaQuick gel extraction kit (Qiagen), and plasmids were purified using the Qiagen QIAprep Spin Miniprep kit. All plasmids were sequence verified by Eurofins Genomics. Recombinant Human histone octamer was purchased from EpiCypher. For visualisation of nucleosomes, ABfinity rabbit monoclonal histone H3 antibody conjugated with Alexa Fluor 647 was used (Thermo Fisher Scientific).

### **Generation of a 1** × **601-pKYB1 vector**

Two separate DNA fragments (Fragment 1 and Fragment 2) were employed to generate a DNA molecule containing one 601-core sequence flanked by 50 base pairs of identical linker DNA. The two fragments were inserted into CIP-treated PciI-digested pKYB1 by Gibson Assembly cloning. In brief, 200 ng of pKYB1 was incubated with 2 units of CIP and 2 units of PciI in a 10 µL volume at 37 °C for 1 hour. Next, 100 ng (18 fmol, 5 µL) of treated pKYB1 and 55 fmol of each fragment were added to 15 µL of 1.3 × Gibson Assembly Master Mix. The sample was incubated at 50 °C for 1 hour. Note that the linker DNA flanking the 601-core is derived from the original 601-linker sequence, as described in Supplementary Methods.

### **Generation of a 2** × **601-pKYB1 vector**

Two separate DNA fragments (Fragment A and Fragment B) were ligated to generate a DNA molecule (Insert 1) containing two 601-core sequences each flanked by 25 base pairs of identical linker DNA. The use of two fragments was necessary because direct synthesis of Insert 1 is challenging owing to its repetitive nature (Supplementary Methods). Each of the two employed fragments contained a single 601-core sequence, flanked on one end by 25 base pairs of linker DNA and on the other end by ten base pairs of 601-linker DNA coupled to a BamHI or BglII restriction site. Prior to ligation, these fragments were digested with BamHI and BglII, respectively. Restriction reactions were performed with 200 ng of DNA fragment and 5 units of enzyme in a 10 µL volume at 37 °C for 1 hour. The ligation reaction was performed with equal amounts of each fragment for 16 hours at 16 °C. The resulting 2 × 601 product (Insert 1) was inserted into CIP-treated PciI-digested pKYB1 by Gibson Assembly cloning as described above using 18 fmol of treated pKYB1 and 55 fmol of Insert 1. The construction of Insert 1 and linker design are detailed in Supplementary Fig. S1 and Supplementary Methods.

### **Generation of a library of 601-based vectors**

A library of plasmids with varying numbers of 601 motifs was produced by first extracting a 1 × 601 or 2 × 601 segment from the 1 × 601-pKYB1 or 2 × 601-pKYB1 vector, respectively. In brief, 4 µg of the relevant vector was digested with 40 units of SpeI and XmaI at 37 °C for 4 hours in a 50 µL volume and products were subsequently gel purified after electrophoretic separation. The resulting segments (Inserts) could then be embedded in either the 1 × 601-pKYB1 or 2 × 601-pKYB1 backbones. In our case, a 4 × 601-pKYB1 vector was constructed using Gibson Assembly, in which the 2 × 601 segment (Insert 2, 417 base pairs) was embedded in a 2 × 601-pKYB1 backbone. The 2 × 601-pKYB1 backbone was obtained by cutting and dephosphorylating the 2 × 601-pKYB1 vector using PciI and CIP, respectively, as described above. The resulting 4 × 601-pKYB1 vector was then used in a similar fashion to obtain plasmids containing eight and twelve 601 repeats (8 × 601-pKYB1 and 12 × 601-pKYB1, respectively). See Supplementary Fig. S5 for a schematic overview of the protocol.

### **Transformations and cell culturing**

Following Gibson Assembly, samples were transformed into NEB 10-beta competent *Escherichia coli* (High Efficiency) cells. In brief, 4.5 µL of the Gibson Assembly mixture (containing 601-based vectors, see above) was added to 50 µL of competent cells on ice. Cells were heat shocked at 42 °C for 30 seconds and then returned to ice. Next, 950 µL of SOC outgrowth medium was added, after which the mixture was incubated at 37 °C for 1 hour, while shaking. After recovery, cells were spread on warm selection plates and incubated overnight. Cells were grown at 37 °C on Lysogeny Broth (LB) agar plates (2%) or cultured in LB. The LB media contained 50 µg/mL kanamycin at all times.

### **Screening of positive clones**

After each transformation, colonies were picked from the LB agar plates and grown overnight. Plasmids were isolated and digested using SpeI in order to screen for successful insertions. Only plasmids containing the 601-based inserts possess a SpeI restriction site, and these plasmids can thus be easily distinguished from unmodified pKYB1. In short, 1 µg of plasmid and 10 units of SpeI were incubated at 37 °C in a reaction volume of 50 µL. The digested product was identified by agarose gel electrophoresis.

### **Preparation of a 12-nucleosome array**

The 12 × 601-pKYB1 plasmid was linearised by EcoRI and biotinylated using previously established protocols^79^. Nucleosome reconstitutions were performed by gradient salt dialysis using a Watson Marlow peristaltic pump, following a procedure described by Kaczmarczyk *et al*.^80^. Samples with increasing octamer:601 motif ratios were used containing 100 ng/µL of the 12 × 601-pKYB1 array (~180 nM of 601 motifs). Competitor DNA of 147 base pairs (Supplementary Table S1) was used in 1:1 mass ratio with the 601 array (100 ng/µL). Reconstituted arrays were stored at 4 °C.

### **Single-molecule experiments**

Experiments were performed using combined dual-trap optical tweezers and confocal fluorescence microscopy in a multi-channel laminar flow cell (C-trap, LUMICKS). Prior to experiments, the flow cell was passivated using a solution of 20 mM Hepes-NaOH pH 7.5, 150 mM NaCl, 0.5% (w/v) casein and 0.01% (w/v) BSA. Following this, the flow cell was flushed with the measurement buffer of 20 mM Hepes-NaOH pH 7.5, 100 mM NaCl, 2 mM MgCl_2_, 0.2% (w/v) BSA and 0.02% (v/v) Tween 20. The presence of BSA in the measurement buffer ensured minimal dissociation/degradation of the nucleosomes from the DNA template^34,81^. Biotinylated nucleosome arrays were tethered between two streptavidin-coated polystyrene beads of diameter 1.76 μm (Spherotech BV) *in situ* and this construct was subsequently moved to a channel containing only measurement buffer. Forces applied to the nucleosome array (via displacement of one of the tethered microspheres) were measured using back focal plane detection of the scattered optical trapping light from the stationary bead using a position sensitive detector. In order to visualise nucleosome positions using immunofluorescent staining, ~1 nM 12-nucleosome array was incubated overnight with fluorescently-labelled anti-H3 antibody (100 µg/mL) at 4 °C in a buffer containing 20 mM Hepes pH 7.5, 100 mM NaCl, 2 mM MgCl_2_, 0.4% (w/v) BSA and 0.02% (v/v) Tween 20. Note that these antibodies contain multiple dyes (typically up to 6), due to primary amine labelling. We therefore use these antibodies as a probe for the presence of histone H3 rather than to quantify the number of nucleosomes present on each array. As a control, we additionally labelled histones with Atto-647N using NHS ester labelling (Supplementary Methods). Each fluorescence image shown in Fig. 4 and Supplementary Figs. S3 and S4 is composed of multiple consecutively recorded line scans along the DNA molecule.

## Supplementary information

Supplementary information.
