# Supplementary material for: Constructing arrays of nucleosome positioning sequences using Gibson Assembly for single-molecule studies
Source: Sci Rep. 2020 Jun 18;10:9903. doi: 10.1038/s41598-020-66259-4 (PMC7303147; doi:10.1038/s41598-020-66259-4)
Supplement: Supplementary file 1 — Supplementary information. [file 41598_2020_66259_MOESM1_ESM.pdf]

Supplementary Information for:

Constructing arrays of nucleosome positioning sequences using Gibson Assembly for single-molecule studies

Dian Spakman[1], Graeme A. King[1,2#*], Erwin J.G. Peterman[1#*] and Gijs J.L. Wuite[1#*]

[1]*Department of Physics and Astronomy, and LaserLaB Amsterdam, Vrije Universiteit Amsterdam, De Boelelaan 1081, 1081 HV, Amsterdam, The Netherlands*

[2]*Current address: Institute of Structural and Molecular Biology, University College London, Gower Street, London WC1E 6BT, UK*

[*]*Correspondence should be addressed to G.J.L. Wuite (g.j.l.wuite@vu.nl), E.J.G. Peterman (e.j.g.peterman@vu.nl) or G.A. King (g.king@ucl.ac.uk)*

[#]*These authors contributed equally*

Supplementary Methods

Design of linker DNA sequences in nucleosome arrays

The linker sequences used in this study are derived from the original 601 linker sequences defined by Lowary and Widom (Supplementary Table S1).[1,2] For example, Insert 1 (Fig. 2) contains three identical linker sequences of 25 base pairs each. These linkers are composed of two ten base pair regions derived, respectively, from the downstream and upstream linker domains of the complete 601 sequence (Supplementary Table S1), fused via a BamHI-BglII site (Supplementary Fig. S1, inset). The sequence of each linker region in Insert 1 is as follows: GCATGTATTGGATCTCGCGGCCGCC, where the bases underlined are associated with the BamHI-BglII ligation. In contrast, Fragments 1 and 2 (Fig. 3) each contain a 50 base pair linker sequence that is composed of two 25 base pair domains derived from the downstream and upstream linker regions, respectively, of the original 601 sequence (Supplementary Table S1). The linker sequence in Fragments 1 and 2 is as follows: GCATGTATTGAACAGCGACCTTGCCGTGATGGACCCTATACGCGGCCGCC.

Choosing the appropriate approach for the design of nucleosome arrays

Directly synthesising repetitive sequences longer than eleven base pairs can be technically challenging, and such long repetitive sequences are typically not available commercially. For this reason, it is only possible to directly synthesise a single 601-core flanked by identical linkers with a maximum of eleven base pairs – much shorter than those found *in vivo* (ranging from ~13–73 base pairs).[3] In order to construct an insert containing two identical 601-core sequences with identical linkers of more than eleven base pairs, such as Insert 1 in Fig. 2, we ligated two smaller fragments together (Fragments A and B, see Methods and Supplementary Fig. S1). These two fragments each contained a 601-core flanked by two 601-linker regions: one of these linkers was of the desired length (> eleven base pairs), and the other (≤ eleven base pairs) was adjoined to a restriction site. In our case, the restriction sites used were BamHI and BglII, respectively (Supplementary Fig. S1, inset). Ligation of these fragments yields a linker sequence of the desired length. Nevertheless, this approach is limited to linker lengths < ~30 base pairs (*i.e.* resulting from two eleven base pair linkers fused via a six base pair BamHI-BglII site). To overcome this limitation, two separate fragments (Fragment 1 and Fragment 2) can instead be used to construct an insert containing a single 601-core flanked by identical linker sequences longer than eleven base pairs (as outlined in Fig. 3).

Labelling of histones with Atto-647N

In order to fluorescently label the histones directly, we covalently linked Atto-647N to free amines of the nucleosomes (to obtain the data presented in Supplementary Fig. S4), using the following protocol. ~1 nM 12-nucleosome array was incubated for 2 hours with 200 μM Atto-647N NHS ester at room temperature in a buffer containing 20 mM Hepes pH 7.5, 100 mM NaCl, 2 mM $MgCl_2$, 0.2% (w/v) BSA and 0.02% (v/v) Tween 20. Atto-647N NHS ester was purchased from ATTO-TEC.

Name	Sequence
Widom 601 sequence [1,2]	CGGGATCCTAATGACCAAGGAAAGCATGATTCTTCACACCGAGTTCATCCCTTATGTGATGGACCCTATACGCGGCCGCC**CTGGAGAATCCCGGTGCCGAGGCCGCTCAATTGGTCGTAGACAGCTCTAGCACCGCTTAAACGCACGTACGCGCTGTCCCCCGCGTTTTAACCGCCAAGGGGATTACTCCCTAGTCTCCAGGCACGTGTCAGATATATACATCCTGT**GCATGTATTGAACAGCGACCTTGCCGGTGCCAGTCGGATAGTGTTCCGAGCTCCC
Fragment A	CCTTTTTACGGTTCCTGGCCTTTTGCTGGCCTTTTGCTCACCCGGGTTCGCCAGTTGGCGTAATAGCGAAGAAGCCCGCACATGTGCATGTATTGGATCTCGCGGCCGCC**CTGGAGAATCCCGGTGCCGAGGCCGCTCAATTGGTCGTAGACAGCTCTAGCACCGCTTAAACGCACGTACGCGCTGTCCCCCGCGTTTTAACCGCCAAGGGGATTACTCCCTAGTCTCCAGGCACGTGTCAGATATATACATCCTGT**GCATGTATTGGATCC
Fragment B	AGATCTCGCGGCCGCC**CTGGAGAATCCCGGTGCCGAGGCCGCTCAATTGGTCGTAGACAGCTCTAGCACCGCTTAAACGCACGTACGCGCTGTCCCCCGCGTTTTAACCGCCAAGGGGATTACTCCCTAGTCTCCAGGCACGTGTCAGATATATACATCCTGT**GCATGTATTGGATCTCGCGGCCGCCCTGGAGAACTAGTTTCTTTCCTGCGTTATCCCCTGATTCTGTGGATAACCG
Fragment 1	CCTTTTTACGGTTCCTGGCCTTTTGCTGGCCTTTTGCTCACCCGGGTTCGCCAGTTGGCGTAATAGCGAAGAAGCCCGCACATGTGCATGTATTGAACAGCGACCTTGCCGTGATGGACCCTATACGCGGCCGCC**CTGGAGAATCCCGGTGCCGAGGCCGCTCAATTGGTCGTAG**
Fragment 2	**CTGGAGAATCCCGGTGCCGAGGCCGCTCAATTGGTCGTAGACAGCTCTAGCACCGCTTAAACGCACGTACGCGCTGTCCCCCGCGTTTTAACCGCCAAGGGGATTACTCCCTAGTCTCCAGGCACGTGTCAGATATATACATCCTGT**GCATGTATTGAACAGCGACCTTGCCGTGATGGACCCTATACGCGGCCGCCCTGGAGAACTAGTTTCTTTCCTGCGTTATCCCCTGATTCTGTGGATAACCG
Sequencing primer	CAGTTGGCGTAATAGCGAAG
Competitor DNA	ATTCATTAATGCAGCTGGCACGACAGGTTTCCCGACTGGAAAGCGGGCAGTGAGCGCAACGCAATTAATGTGAGTTAGCTCACTCATTAGGCACCCCAGGCTTTACACTTTATGCTTCCGGCTCGTATGTTGTGTGGAATTGTGAGC

Supplementary Table S1. Sequences of all DNA fragments used in this study. The 601-core sequence of 147 base pairs, as defined by Lowary and Widom,[1,2] is shown in bold. Underlined bases correspond to DNA linker sequences. The full-length linker sequences defined by Lowary and Widom consist of (i) 80 base pairs upstream of the 601-core and (ii) 55 base pairs downstream of the 601-core, as shown in the first row of this table. Linker sequences associated with Fragments A, B, 1 and 2 are derived from the above full-length linker sequences. Bases in grey block correspond to restriction sites.

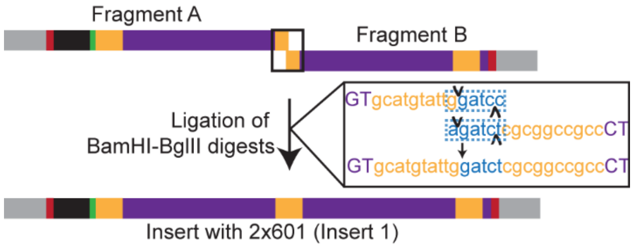

Supplementary Figure S1. Generation of a 2×601 construct (Insert 1, see Figs. 1 and 2) for sequential Gibson Assembly cloning. Here two fragments (Fragment A and Fragment B) are ligated together. Each fragment contains a single 601-core (purple) that is flanked immediately on one end by 25 base pairs of linker DNA (longer yellow region) and on the other end by ten base pairs of 601-linker DNA (shorter yellow region). These ten base pairs are extended respectively to form BamHI or BglII restriction sites (highlighted by the blue dashed boxes in the inset). Ligation of Fragments A and B following BamHI-BglII digestions therefore results in an array of two 601-core sequences, each flanked by 25 base pairs of linker DNA. Note that the bulk of the linker DNA flanking each 601 motif here is derived from the original 601-linker sequence (as defined by Lowary and Widom)[1,2] with the exception of five base pairs (as shown in blue in the inset and described in Supplementary Methods). A number of regulatory sequences are additionally present on each end of the 2×601 insert, required for further Gibson Assembly reactions (shown here in grey, black, green and red, and described in detail in Fig. 1B).

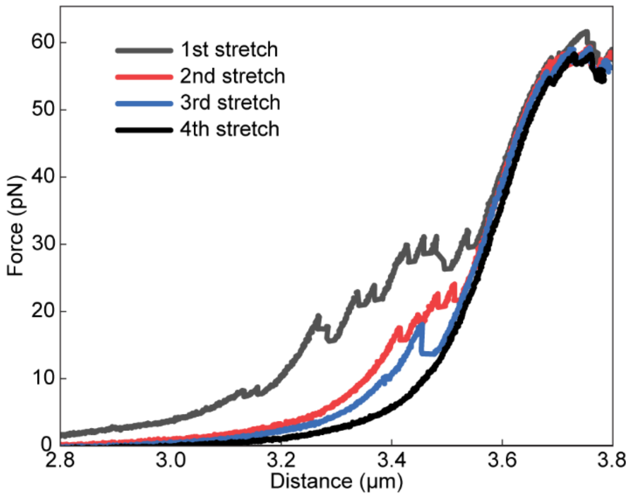

Supplementary Figure S2. **Repeated stretching of nucleosome arrays hinders nucleosome re-wrapping.** Sample force-extension curves showing repeated stretch-relax cycles for the 12×601-pKYB1 nucleosome array. The substrate was stretched at a modest rate of 20 nm s^{-1} to ~60 pN before the extension was reduced and the molecule re-stretched. The first stretch (grey) displays the characteristic force ruptures associated with nucleosome unwrapping.[4-7] The second stretch (red) shows fewer force ruptures indicating only a small number of nucleosomes had re-formed after the first stretch. The third stretch (blue) shows even fewer force ruptures, and thus very little nucleosomes had re-formed after the second stretch. The fourth stretch (black) did not exhibit any force ruptures. This is consistent with previous studies that have reported little or no nucleosome re-wrapping after repeated stretching of a nucleosome array.[4-7]

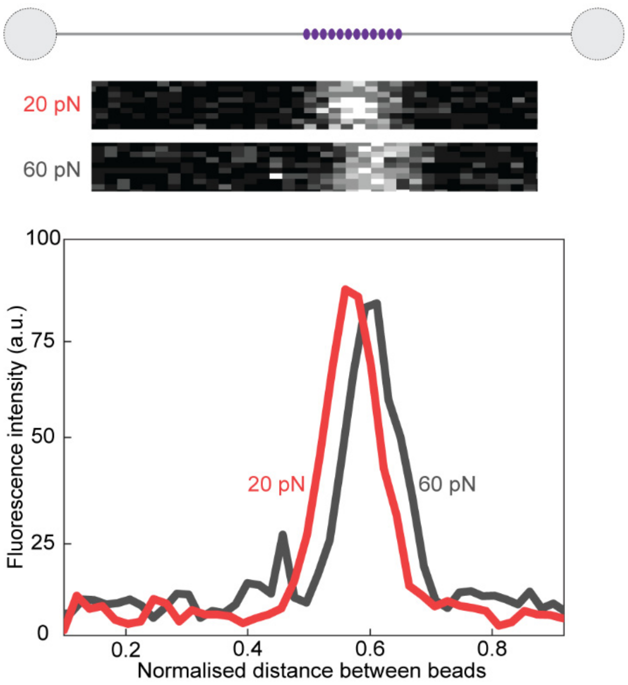

Supplementary Figure S3. Fluorescence intensity associated with H3-antibody-labelled nucleosome arrays does not change significantly between 20 pN and 60 pN. The plot shows a comparison of the total fluorescence intensity along the 12×601-pKYB1 nucleosome array at 20 pN and 60 pN, obtained during the first and second stretch, respectively. The corresponding fluorescence images (reproduced from those in Fig. 4C) are shown above. There is little change in the fluorescence intensity between the two images, indicating that antibody-labelled H3 histones remain bound to the DNA between these two forces, and even at forces ≥ 60 pN (Fig. 4D).

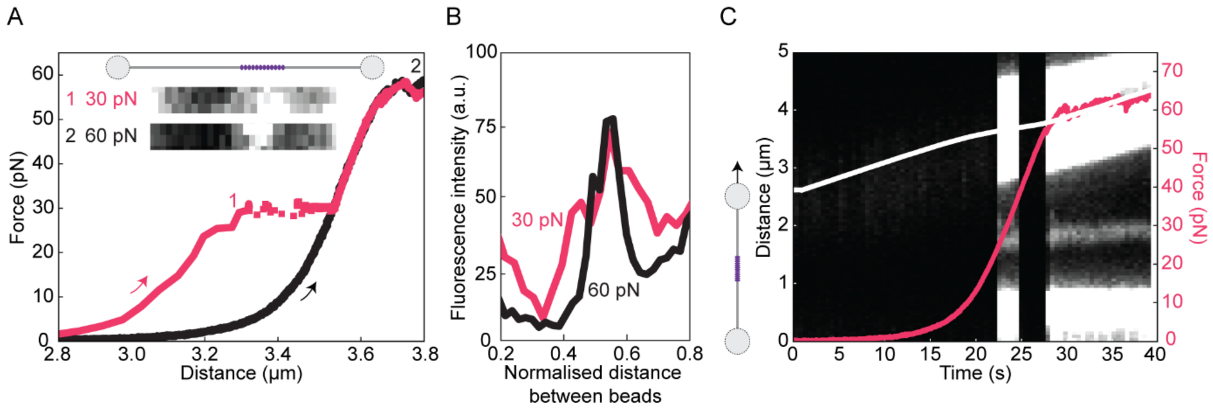

Supplementary Figure S4. Fluorescence from Atto-647N-labelled nucleosome arrays as a function of force. (A) Sample force-distance curve of the 12×601-pKYB1 nucleosome array (pre-incubated with Atto-647N NHS ester). Here, the substrate was first stretched to 30 pN (1), at which point a fluorescence snapshot was recorded (inset, upper image). The molecule was subsequently held at 30 pN until all nucleosomes had unwrapped and then stretched further to the onset of overstretching (red trace). Following this, the DNA extension was decreased (data not shown) before being re-stretched (black trace). No ruptures were observed during this re-stretch, indicating that no nucleosome re-wrapping had occurred after the first stretch-relax cycle. However, a fluorescence snapshot recorded after the second stretch at 60 pN (2) reveals that histones are still present (inset, lower image). This mirrors the observations for antibody-labelled nucleosomes shown in Fig. 4C. **(B)** Comparison of the fluorescence intensity (from Atto-647N-labelled histones) along the length of the DNA substrate at points (1) and (2) in panel A. This comparison indicates that many histones stay bound to the DNA even after no nucleosome re-wrapping could occur. These histones are most likely histones H3 and H4, owing to the fact that the H2A/H2B dimers have been suggested to dissociate at forces < 30 pN.[8,9] Note that we are unable to quantitatively compare the Atto-647N fluorescence intensity at forces < 30 pN due to the significant background fluorescence when the beads are close together. **(C)** Sample kymograph showing Atto-647N-labelled histones bound to the 12×601-pKYB1 construct as a function of time as it is stretched at a speed of 50 nm s^{-1}. The corresponding force and extension are overlaid on the kymograph in red and white lines, respectively. Note that prior to this measurement, the DNA substrate was stretched several times until no nucleosome re-wrapping was observed. In order to minimise bleaching, the fluorescence excitation source was only turned on at certain points during stretching: first, when the substrate was at ~30 pN, and second, when the DNA was being overstretched (> 60 pN). Similar results were obtained for all molecules studied in this way ($N > 10$).

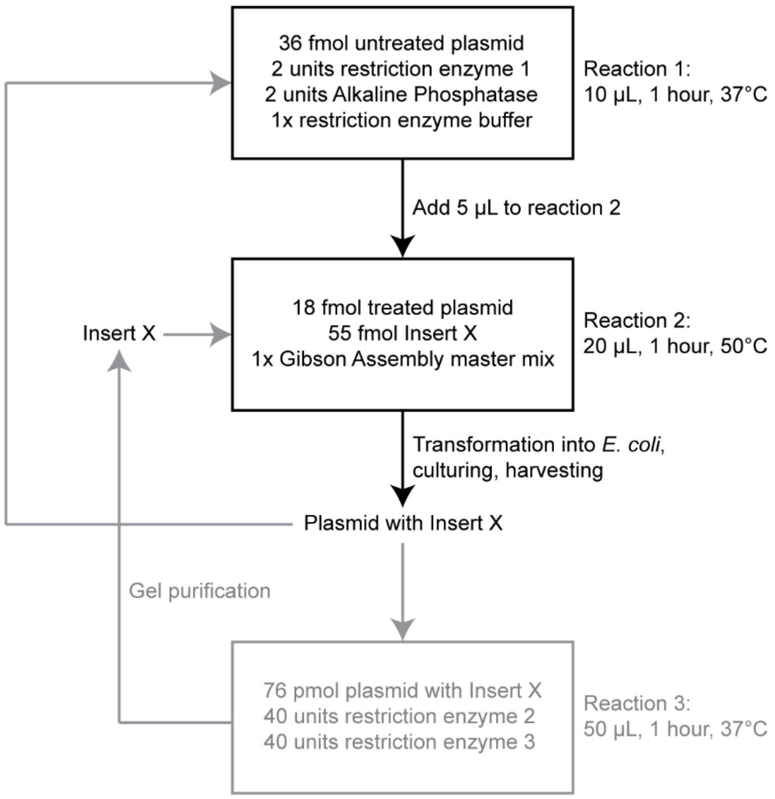

Supplementary Figure S5. Protocol and workflow for the generation of arrays of sequence motifs using the methods presented in Figs. 1 and 3. A suitable plasmid is first linearised using restriction digestion (Reaction 1). Next, an insert (Insert X) is embedded into the above substrate via a Gibson Assembly reaction to yield a plasmid containing Insert X (Reaction 2). Insert X here corresponds to an n×601 insert. The plasmid containing Insert X is then subjected to two parallel digestion reactions. In one reaction, the product is digested at two sites (Reaction 3, *c.f.* Fig. 1) to yield a new Insert X. In the second parallel reaction, the product is digested at a single restriction site (Reaction 1, *c.f.* Fig. 1) to yield a linearised vector for a second Gibson Assembly reaction to generate a plasmid containing two tandem repeats of Insert X. In this way, a library of plasmids with defined arrays of a specific motif can be generated.

References

[1] Lowary, P.T. & Widom, J. New DNA sequence rules for high affinity binding to histone octamer and sequence-directed nucleosome positioning. *J. Mol. Biol.,* **276**, 19–42 (1998).

[2] Flaus, A. Principles and practice of nucleosome positioning *in vitro. Front. Life Sci.*, **5**, 5–27 (2011).

[3] Perisic, O., Collepardo-Guevara, R., & Schlick, T. Modeling studies of chromatin fiber structure as a function of DNA linker length. *J. Mol. Biol*., **403**, 777–802 (2010).

[4] Brower-Toland, B.D. *et al.* Mechanical disruption of individual nucleosomes reveals a reversible multistage release of DNA. *Proc. Natl. Acad. Sci. U.S.A*., **99**, 1960–1965 (2002).

[5] Bennink, M.L. *et al.* Unfolding individual nucleosomes by stretching single chromatin fibers with optical tweezers. *Nat. Struct. Mol. Biol*., **8**, 606–610 (2001).

[6] Pope, L.H. *et al*. Single chromatin fiber stretching reveals physically distinct populations of disassembly events. *Biophys. J*., **88**, 3572–3583 (2005).

[7] Gemmen, G.J. *et al*. Forced unraveling of nucleosomes assembled on heterogeneous DNA using core histones, NAP-1, and ACF. *J. Mol. Biol.,* **351**, 89–99 (2005).

[8] Meng, H., Andresen, K. & van Noort, J. Quantitative analysis of single-molecule force spectroscopy on folded chromatin fibers. *Nucleic Acids Res*., **43**, 3578–3590 (2015).

[9] Claudet, C., Angelov, D., Bouvet, P., Dimitrov, S. & Bednar, J. Histone octamer instability under single molecule experiment conditions. *J. Biol. Chem*., **280**, 19958–19965 (2005).
